# Effect of pitch range on dogs’ response to conspecific vs. heterospecific distress cries

**DOI:** 10.1038/s41598-021-98967-w

**Published:** 2021-10-05

**Authors:** Holly Root-Gutteridge, Victoria F. Ratcliffe, Justine Neumann, Lucia Timarchi, Chloe Yeung, Anna T. Korzeniowska, Nicolas Mathevon, David Reby

**Affiliations:** 1 School of Psychology, University of Sussex, Falmer, East Sussex BN1 9QH UK; 2 Defence Science and Technology Laboratory, Salisbury, Wiltshire SP4 0JQ UK; 3 Equipe de Neuro-Ethologie Sensorielle ENES/CRNL, University of Lyon/Saint-Etienne, CNRS UMR5292, INSERM UMR_S 1028, Saint-Étienne, France

**Keywords:** Animal behaviour, Social evolution

## Abstract

Distress cries are emitted by many mammal species to elicit caregiving attention. Across taxa, these calls tend to share similar acoustic structures, but not necessarily frequency range, raising the question of their interspecific communicative potential. As domestic dogs are highly responsive to human emotional cues and experience stress when hearing human cries, we explore whether their responses to distress cries from human infants and puppies depend upon sharing conspecific frequency range or species-specific call characteristics. We recorded adult dogs’ responses to distress cries from puppies and human babies, emitted from a loudspeaker in a basket. The frequency of the cries was presented in both their natural range and also shifted to match the other species. Crucially, regardless of species origin, calls falling into the dog call-frequency range elicited more attention. Thus, domestic dogs’ responses depended strongly on the frequency range. Females responded both faster and more strongly than males, potentially reflecting asymmetries in parental care investment. Our results suggest that, despite domestication leading to an increased overall responsiveness to human cues, dogs still respond considerably less to calls in the natural human infant range than puppy range. Dogs appear to use a fast but inaccurate decision-making process to determine their response to distress-like vocalisations.

## Introduction

In many mammals, distress cries are often produced by isolated, discomforted, endangered, or hurt infants and have the vital function of eliciting caregiving behaviour from the listener, often the parents^1–4^. Cries signal that potential or actual harm is occurring, and as the causes of the cry can be both dangerous (e.g., a predator is present) and time critical (e.g., a predator attack may be imminent), demands quick but potentially costly reactions from helpers^5^. Across vertebrate taxa, infant distress cries tend to share acoustic features such as a high pitch, high intensity and the presence of nonlinear phenomena^5^, while the structure is commonly a simple slope^6^, suggesting that cry production may be constrained by the weak motor control infants have over their vocal folds. Furthermore, distress cries represent an important source of information to the listener as they can convey informational cues regarding the speaker’s species^7^, identity^8–10^, sex^11^, age^9,12,13^, and health^14,15^, as well as signalling behavioural context^5,7,16^. Bremond^17^ determined that some of these call features would act as “rejection markers”, which the listener uses to refuse to respond to signals, e.g. if the call’s frequency is too far outside of the species-specific range, it may be ignored. These rejection markers are critical to the listeners’ response, and can be separated from the irrelevant features such as call duration, but do not have to be species-specific^6,17^.

Because crying is energetically costly and can increase the risk of predation, it has been proposed that it should be an honest signal of need^16,18^, and listeners’ responses are expected to be shaped by the information in the cry (e.g., species)^3^. Efficient caregiving is critical to reproductive success, and as such there is a conflict between the risk of failing to respond to own offspring and that of responding to non-specific offspring^18^. Indeed, caregiving behaviour is energetically costly and can negatively affect the caregiver’s own fitness and reproductive success^19,20^, so it could be expected that animals will at least limit their response to their own species. In humans, it has been proposed that crying may therefore be a manipulative, graded signal, functioning to increase caregiving from the listener^21^, while acoustically abnormal infant cries can indicate serious distress or pathologies^16^.

While perception of the emotional valence and arousal of calls may be shared across species^22,23^, caregiving responses are expected to remain species-specific^5^. Yet, it has been demonstrated that both female mule deer (*Odocoileus hemionus*) and white tailed deer (*O. virginianus*) show similar behavioural responses to conspecific and hetero-specific distress cries which share the range of the fundamental frequency or pitch of cries^3,24^. This suggests that calls are sufficiently close acoustically to be accepted as functionally equivalent and elicit the same behaviour^3,24^. However, a study investigating the spontaneous ability of naïve human adults to assess the emotional content of vocalizations in closely related ape species, including bonobos (*Pan paniscus*) and chimpanzees (*P. troglodytes*), showed that listeners responses were strongly biased by basic frequency differences^25^, indicating that in the absence of exposure and/or training, the interspecific relevance of emotional signals can be limited by simple acoustic scaling differences^25^.

Domestic dogs are potentially special because a long history of domestication^26^ has resulted in close, affectional, social bonds between dogs and humans^27,28^, with dogs attending to both adults and children^29^. Dogs have shown themselves to be sensitive to a wide range of human cues^30–37^ and to attend more to adult humans when in distress than when relaxed^38^. Furthermore, dogs show a similar physiological response to humans when hearing human infant distress cries, which increases their cortisol levels, a marker for stress^39^. However, while dogs are often said to respond empathetically to distress in adult humans^38,40–42^, it is not known whether domestication has affected their responses to human infants.

Like humans, dogs give calls from birth, including frequency modulated and harmonically structured cries, whose fundamental frequency (*f0*) ranges from 30–13,000 Hz, decreasing with age^43^. Dogs of both sexes attend to puppy cries by orienting and moving towards the loudspeaker from where they were emitted^44^. Human vocalisations are also familiar and relevant to domestic dogs^45^ and there is evidence that dogs can recognise and respond to their emotional valence^30,32,46^, particularly if the valence is negative, as in distress sounds^38,46^. In fact when dogs hear baby cries, they are known to show increased levels of cortisol and to show submissive behaviours: they put their ears flat and back, lower the tail, head, and body, all characteristics of an aversive response^39^. Interestingly, despite differences in parental investment, the dogs’ sex does not affect their level of attention to cries from either puppies or human babies^44,47^.

Here, we investigate whether adult male and female dogs respond differently to conspecific puppy distress cries vs. heterospecific human infant cries. Using resynthesis, we also test the effect of the cry frequency range (conspecific vs. heterospecific) of the *f0* on their response. We predict that, overall, dogs are more likely to respond to puppy than to human baby calls. Moreover based on prior observations^38,44,47^, we do not expect to find differences between male and female dogs when responding to human baby cries, but to find such differences when responding to puppy cries. We also predict that, as seen in other species^1,24^, the frequency range (conspecific vs. heterospecific) of the calls should influence dogs’ responses independent of call origin (conspecific vs. heterospecific), with dogs giving stronger responses to conspecific and heterospecific calls modified to align the fundamental frequency range with conspecific values. Finally, following published observations that adult humans are able to discriminate between levels of distress in human babies on the basis of cry acoustic, we explore whether dogs respond differently to cries from babies that are experiencing acute pain from vaccination vs. cries from babies experiencing mild discomfort from being bathed by their parents^25^. We predict that cries recorded during vaccination should elicit stronger responses in dogs.

## Results

### Descriptive variables of call fundamental frequency

The mean, maximum, minimum, range, and slope of the fundamental frequency (F0) varied significantly between puppy and baby cries (p < 0.001, see Electronic Supplementary Materials (ESM) for details), as did the number of voice breaks (F_1,16_ = 10.325, p = 0.005), while the number of large inflections and coefficient of variation did not differ (p > 0.1). The cause of distress in babies, e.g. vaccine or bath, did not significantly affect any F0 variable (t-test: p > 0.1 for all variables, see ESM for details).

### Effect of Caller species (con- vs hetero-specific) and Call frequency (con- vs hetero-specificon dogs’ reactions to distress cries

**Figure 1:**
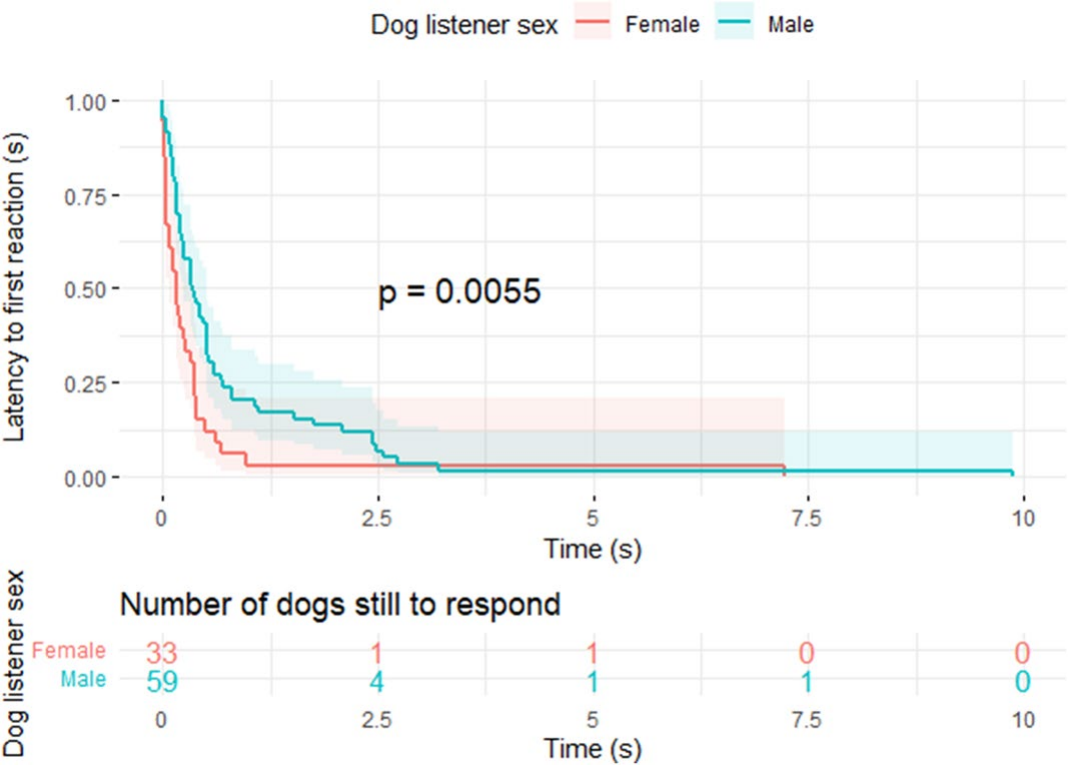
Survivorship plot for latency to first attention for male and female dogs, with time in seconds, p = 0.006. The survivorship plot maps how long it takes to reach a completed response (or time for the trial elapses) and compares this across factors. Male dogs have a longer latency to react than female dogs.

We used survivorship analysis to analyse the latency to reaction, e.g., the time spent before reacting to the stimulus. Neither Caller species (con- vs hetero-specific, p = 0.19), Call frequency (con vs heterospecific, p = 0.81), nor the interaction of Caller species and Call Frequency (p = 0.78) affected the latency to first reaction. However, the dog-listener Sex did affect latency to first reaction (p = 0.006), with male dogs responding more slowly to the calls than females (median response time 0.16 s for females, 0.35 s for males, Fig. 1).

**Figure 2:**
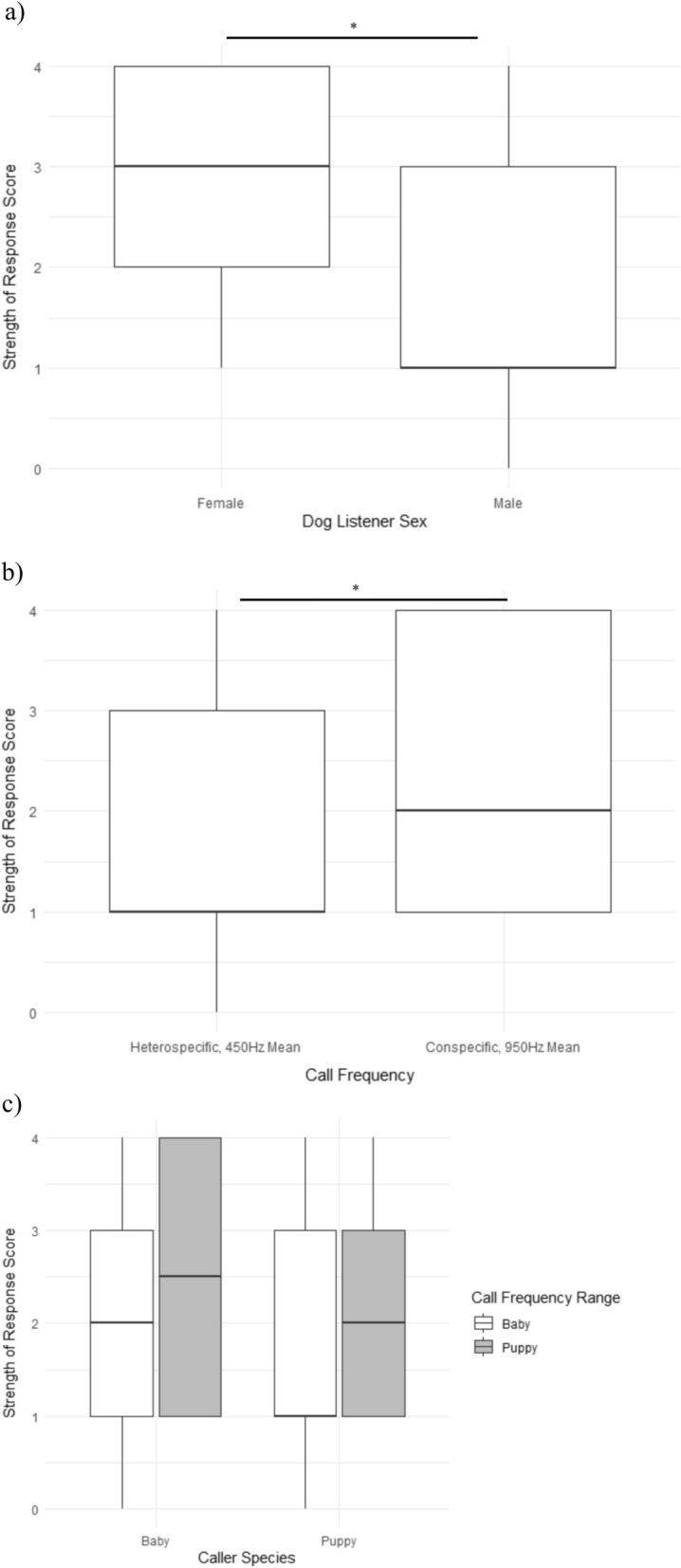
Boxplots of Score of intensity of dogs’ response to distress cries from human babies and puppies in either their natural or modified frequency (950 Hz or 450 Hz) by (**a**) Sex, (**b**) Call frequency, and (**c**) the interaction between Caller Species and Call frequency. Male dogs’ reactions were scored lower than female dogs’ (F_1,23_ = 5.5, p = 0.027), while reactions to cries in the conspecific range were given higher scores than cries in the heterospecific range (F_1,67_ = 4.3, p = 0.042), regardless of Caller species (F_1,67_ = 5.5, p = 0.488). There was no significant interaction between Caller species and Call frequency (F1,68 = 0.1, p = 0.738).

When we tested the effects of Caller species, Call frequency, their interaction, and dog listener Sex on response strength scores with dog listener identity included as a random effect in a Linear Mixed Model (LMM, Table 1), we found that Caller species did not affect the Response score either by itself or in interaction with Call frequency, while dog Sex and Call frequency both did (p < 0.05, Table 1, Fig. 2). Overall, female dogs gave significantly stronger responses than male dogs (mean female score = 2.60, std. error = 0.25, vs mean male score = 1.87, std. error 0.19, Fig. 2a) and conspecific-frequency cries elicited higher scores than heterospecific-frequency cries (mean conspecific-frequency score = 2.48, std. error = 0.20, vs mean heterospecific-frequency score = 1.99, std. error 0.20, Fig. 2b).

**Table 1:** Linear mixed model results for response scores for (a) both puppy and baby trials together and (b) baby cry trials only.

Dataset	Fixed effect	Numerator df	Denominator df	F	Sig.
Scores in response to both baby and puppy cries	Intercept	1	23.4	207.2	< 0.001
	Caller species	1	66.6	0.5	0.488
	**Call-frequency**	**1**	**66.6**	**4.3**	**0.042**
	**Dog-listener sex**	**1**	**23.4**	**5.5**	**0.027**
	Caller species * call-frequency	1	67.9	0.1	0.738
Scores in response to only baby cries	Intercept	1	42	160.406	< 0.001
	Call-frequency	1	42	2.463	0.124
	Call-stimulus-type	1	42	0.998	0.323
	**Dog-listener sex**	**1**	**42**	**6.898**	**0.012**
	Call-frequency * call-stimulus-type	1	42	0.398	0.531

Significant results (p < 0.05) are marked in bold.

### Effect of human baby cries recording context (bath vs. vaccination) on dogs’ reactions

To determine whether dogs responded differently to the babies’ cries depending on the type of eliciting stimulus (Call stimulus-type: pain or discomfort), we tested the effects of Call stimulus-type on latency and reaction strength. Survivorship analysis showed no effect on dogs’ latency to react (p = 0.58). To test the effect of cry type (vaccination or bath) on dogs’ response strength, we ran a LMM with Response scores as the dependent variable and Call-stimulus-type, Call frequency (con- vs heterospecific), their interaction, and dog-listener Sex as fixed factors and dog identity as the random effect. Only Sex was found to have a significant effect (F_1,42_ = 6.898, p = 0.012, Table 2) with female dogs reacting more strongly than males, showing that overall, dogs were not affected by the context in which the baby had been crying.

**Table 2:** Definitions for ordinal scale of attentional responses to the stimuli where 0 is no visible reaction and 4 is strongest reaction.

Ordinal scale for response scores	Head turn	Posture change	Movement from starting point	Approach basket	Interact with basket
0	N	N	N	N	N
1	Slow/incomplete	N	N	N	N
2	Fast/complete	Y	N	N	N
3	Fast/complete	Y	Y	Incomplete/slow	N
4	Fast/complete	Y	Y	Fast/complete	Y

## Discussion

Here we tested whether dogs would respond differently to the distress cries of human babies and puppies, emitted from a loudspeaker in a covered basket, and if so, whether this was driven by the fundamental frequency of the call. We found that dogs paid more attention to the playback when the distress cry shared the 950 Hz frequency range with puppies, regardless of whether it was a human baby or a puppy crying, while the reason for the cry, whether pain of vaccination or discomfort from being bathed, had no effect*.* Dogs showed greater attention to the playback when the frequency of the human baby cry was shifted to the conspecific range (i.e. mean F0 950 Hz), and less to the puppy cry when it was shifted to the heterospecific range (i.e. mean F0 450 Hz). This suggests that the frequency range is a key factor affecting the intensity of the response to con- and hetero- specific calls, and thus a potential rejection marker. This is in agreement with previous findings in deer, where frequency range rather than species (con- vs. heterospecific) determined attentional response^1,24^, and shows that call frequency can to some extent override other acoustic features, such as the number of voice breaks and contour slope.

The effects of domestication in dogs have been well-documented^48^ while the perception of emotion across species has been receiving increasing attention, including animals’ sensitivity to human emotional cues in both voices and faces^49,50^. There is also increasing evidence that the features which encode emotional valence are conserved across mammalian species and that emotional perception is similarly shared^22,23,51^. Here we found that dogs’ overall response depended on the cry frequency range, not the species of caller. Some of the puppy cries also include a second fundamental frequency, referred to as *g0*^52–55^, and present between 2.95 and 10.43 kHz, while a third ultra-high frequency H0 has recently been identified in adult domestic dog whines at 9.9–23.26 kHz^54^. In this study, we focused on comparing responses to the fundamental frequency of puppy whines vs. human baby cries. A previous study showed that dogs displayed similar responses to playbacks of adult human cries and dog isolation whines^46^. Surprisingly, we also found no difference in attentiveness to distress cries from babies in pain rather than mild discomfort, despite the fact that the babies’ parents are capable of distinguishing the two^56^. This may either be a consequence of the dogs’ lack of experience with babies or of their lack of ability to discriminate between the two call types when the F0 variables did not differ. Further investigations could explore whether modifying other features known to encode emotional valence and arousal would influence response to calls, in particular, the jitter, harmonics-to-noise ratio, and inclusion of non-linear phenomena.

Responding to distress cries may involve a fast-but-inaccurate decision making process, where the speed of response is more critical than correctly identifying one’s own infant when the cost is potentially injury or death to the offspring^3,57^. Here, the shared frequency range of natural-puppy and modified-baby cries was sufficient to elicit similar attention to the caller regardless of species. This supports the theory that species should respond when call-frequency range is shared as the potential costs of a false-positive response are outweighed by the costs of not responding^57^*.* We found that dogs paid less attention to cries out of their natural frequency range, fitting with a previous result that dogs respond more to higher frequency puppy cries^44^, and paralleling human perception of frequency and arousal in distress cries as increasing together and therefore eliciting stronger responses^25^. Alternatively, as dogs are known to judge the body size of other dogs from their growls^58^, and fundamental frequency is known to encode body size in mammals^59–61^, the dogs in our study may have estimated the body size of the caller from the frequency of the cries and shown less attention when they perceive them to be larger, as potentially less in need of defence from predators, or even posing a potential threat to the listening dog itself.

We found a strong effect of receiver sex on responsiveness, as female dogs paid more attention to the playback and responded faster than male dogs across all stimuli. This contrasts with previous results that showed male and female dogs were equally responsive to distress cries^44,47^. However, our results fit with the fact that male dogs invest less parental care in their offspring^62^ than females do, thus females might be under stronger selective pressures to respond quickly to distress cries. Further research should examine why the sex difference was found here but not in other studies and to explore the potential effects of experience with puppies and babies, neuter status, and sociability on the dogs’ responses. While our demographic survey recorded the dogs’ neuter status and experiences, there was not enough variance in our sampled population to explore their effects in the analysis.

Overall, our results showed that dogs respond equally to baby and puppy cries in their natural frequency ranges, similar to results for adult humans and dogs^46^. However, dogs produced a stronger attentional response to cries in the conspecific frequency range than in the heterospecific range, supporting the hypothesis that frequency range is critical to distress cry response. Species of caller and reason for cry did not alter the dogs’ response, though the dogs’ sex was influential.

## Methods

Vocalisations were collected from (a) 12 human infants aged approximately 2 months which had been collected for a previous paper^56^, and (b) 6 Labrador puppies aged approximately 9 weeks old which were sampled from YouTube videos. The puppies had been recorded producing distress cries when alone and probably not in pain by their owners. The babies were recorded crying during routine care and were in (a) discomfort but not pain when placed into a bath of warm water, or (b) pain following their vaccination injections in a doctor’s office^56^. Initially, 18 stimuli were created from each of the sources: 6 presenting a puppy in isolation, 6 presenting a baby crying when being bathed, and 6 presenting a baby crying after vaccination. Each infant was only sampled once. Each stimulus presented 5 s of distress crying from one of six individuals followed by 10 s of silence, with amplitude of all files normalised to − 9 dB using Audacity. The sample rate for each file was set to 44,100 Hz and bit rate to 16-bit. The fundamental frequency of each file was analysed in Praat^63^ and the mean, maximum, minimum, range, coefficient of variation, number of large inflexions of more than 25 Hz per time step, number of voice breaks, and mean absolute slope were measured. Each of the stimuli was then modified using the Change Gender function in Praat, so that the fundamental frequency of the cries was shifted to match that of the other species, either up to 950 Hz to match puppies or down to 450 Hz to match human babies. Formant frequencies and other factors were not changed. This resulted in a total of 36 stimuli, 18 natural and 18 modified sounds.

A total of 40 adult dogs participated in the study, with 15 dogs removed because of technical difficulties, distractions, or background noise. A total of 92 trials from 25 dogs (9 females, 16 males; aged 10 months to 12 years old, mean = 5.19 years old) representing 19 breeds and mixed breeds were retained (see ESM for demographic details). Owners were also surveyed regarding the dogs’ neuter status and previous experience with (a) babies and (b) puppies. However, as only 1 of the 9 female dogs was intact and only 10 dogs had extensive experience with babies and 4 had extensive experience with a puppy, we did not include the neuter status or prior experience in the analysis. These should be considered in future research.

**Figure 3:**
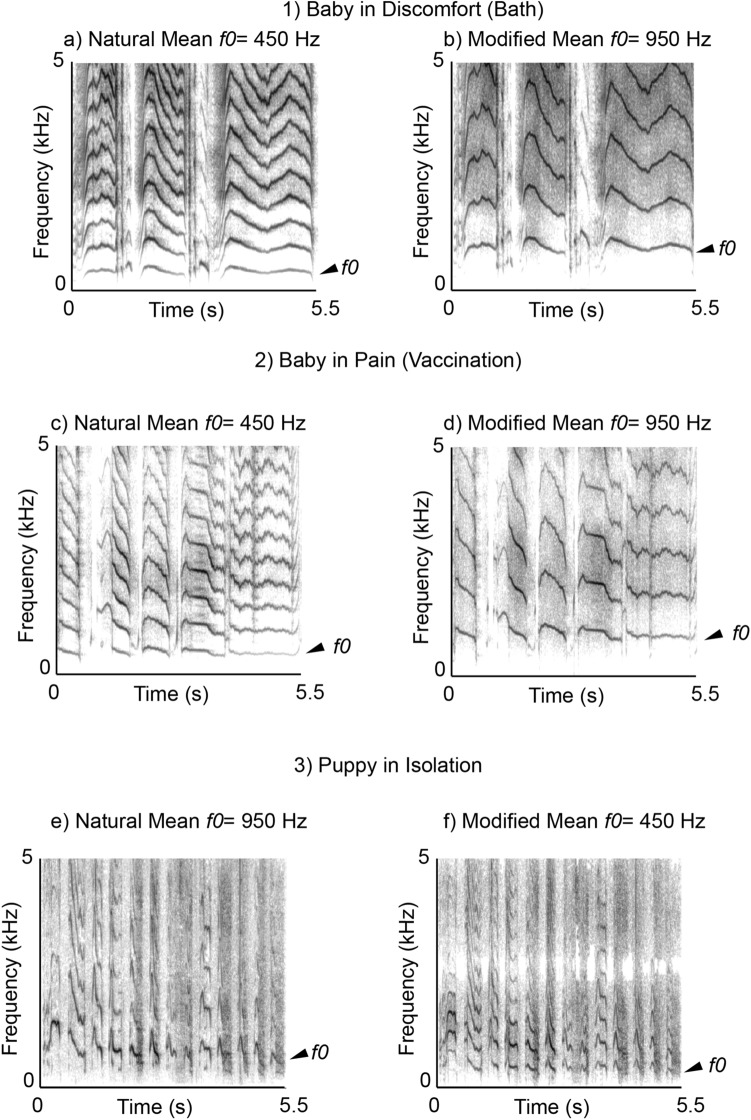
Spectrograms exemplifying the different cry types used. Each cry was resynthesised and presented at both the typical human-baby frequency range of mean = 450 Hz, which the dogs should consider heterospecific, and at the typical puppy frequency range of mean = 950 Hz, which the dogs should consider conspecific: (**a**) the distress cry from a human baby in a bathtub at natural, 450 Hz mean pitch (discomfort-baby heterospecific 450 Hz), (**b**) the same baby cry modified to 950 Hz mean pitch (discomfort-baby conspecific 950 Hz), (**c**) a second baby’s cry following vaccination at natural, 450 Hz mean pitch (baby-vaccination heterospecific 450 Hz), (**d**) the same cry modified to 950 Hz mean pitch (baby-vaccination conspecific 950 Hz), (**e**) approximately 9-week old puppy crying while isolated, presented at modified, 450 Hz mean pitch (puppy-heterospecific 450 Hz), and (**f**) the same puppy cry presented at natural, 950 Hz mean pitch (puppy-conspecific 950 Hz). The spectrogram settings were: FFT = 1024, viewing range 0–5000 Hz, window length = 0.05 s, time step = 0.005 s).

All dogs were tested outside at either Stanmer Park or Falmer Campus, Sussex, and accompanied by their owners at all times. Each dog heard two cries from babies and two cries form puppies, presented as four different types of stimuli: the baby cries in heterospecific frequency range (baby-vaccine-natural or baby-discomfort-natural), the baby cries in conspecific frequency range (baby-vaccine-modified or baby-discomfort- modified), a puppy cry in conspecific frequency range (puppy-natural), and a puppy cry in heterospecific frequency range (puppy-modified). See Fig. 3 for example spectrograms. The dogs only heard babies in discomfort or babies after vaccination, thus four-stimuli were heard by each dog and the order of presentation was counter-balanced across subjects. The amplitude of all recordings was normalised in Audacity. The stimuli were presented from a single JBL Bluetooth speaker that was set to conversational volume (approx. 70 dB measured at 1 m distance), with playback controlled on the experimenter’s phone. The speaker was positioned in a basket with a blanket over the opening, to create the illusion that a baby or puppy might be inside, and the basket was placed 5 m to one side of the dog, counterbalanced across subjects. The dogs were positioned by their owners at 90° to the basket and were free-ranging during the tests. The dogs’ reactions were filmed on two camcorders positioned on tripods. The position of dog and basket were rotated for each trial around a square design.

Videos were assessed before coding and discarded if the dog was distracted during trials by non-stimulus sounds or events (n = 63).

Prior to analysis, the audio tracks were removed from the videos, with the stimulus sound replaced by a sound effect to cue the observer as to the start of the sound. Coded titles were then assigned to each file to hide the trial number and stimulus sound. Then, all videos were coded blind in Sportscode Gamebreaker 11 (Sportstec, Warriewood, NSW, Australia) by HRG, with 20 videos (22%) second-coded by ATK. Agreement was measured with Cronbach’s alpha (reliability set to > 0.8 out of 1), calculated in SPSS v. 25 (SPSS Inc., Chicago, IL., USA). 0.97 was achieved for ordinal scores, where > 0.8 is considered excellent.

### Measuring latency to and strength of response

The latency to reaction was measured as the time between the initial onset of the distress cry and the behavioural response by the dog. An ordinal score coding system based on Fukuzawa et al. ^64^ was used to quantify the strength of the dogs’ response to the stimuli, with criteria given in Table 2. The dog’s reaction in each trial was given a single Score based on these criteria. See Electronic Supplementary materials for examples of a reaction where the same dog gave reactions which were scored with either a 1 (filename Emma_Terrier_3.mp4) or a 4 (filename Emma_Terrier_1.mp4).

### Effect of Caller species (con- vs hetero-specific) and Call frequency (con- vs hetero-specific) on dogs’ reaction to distress cries

To explore how Caller Species, Call Frequency, and dog-listener Sex may have affected latency to reaction, survivorship plots were created using the “survival” package in R^65^. Four plots were created: Call Frequency, Caller Species, and dog-listener Sex modelled separately and a model which included both Call frequency and Caller species. Due to limited sample size, we did not explore their interaction with Sex.

To explore the effect of Caller species and Call frequency, their interaction and dog Sex on Response score, a linear mixed effect model (LMM) with restricted-maximum likelihood estimation with 1000 times bootstrapping (performed in SPSS) was fitted. Dog-identity was included as a random effect to account for considerable between-individual variation. As we aimed to maximise the demographic characteristics of sampled dogs, e.g. sex, age, neuter status, and breed, the sample size was not large enough to allow for further interactions or independent variables to be explored.

### Effect of human baby cries recording context (bath vs. vaccination) on dogs’ reactions

To determine whether the dogs responded differently to the babies’ cries depending on eliciting stimulus (pain or discomfort), the latency to reaction and Response score were compared for vaccination vs bath trials. Latency was again modelled in a survivorship plot of Call-stimulus-type (vaccination vs. bath) with p < 0.05.

To test difference in Response scores, a bootstrapped LMM was fitted to the Scores for baby cry trials only with the fixed effects of Call stimulus-type (vaccination or bath), Call frequency (con- or heterospecific), their interaction, and dog-listener Sex, and with dog-identity as a random effect.

### Ethics approval

The infant cry research reported in the present paper was reviewed and approved by the Ethical Committee of the University Hospital of Saint-Etienne (Comité d’Ethique du CHU de Saint- Etienne and the Commission Recherche de Terre d’éthique; Institutional Review Board: IORG0007394). The project reference is IRBN672015/CHUSTE.

Ethics for the experiment with the dogs were granted by the Animal Welfare Ethical Review Board (AWERB) at the University of Sussex under permit number ARG/07/02. The dogs were accompanied by their owners at all times and participation was completed within 30 min. All methods were carried out in accordance with relevant guidelines and regulations.

## Supplementary Information

Supplementary Information 1.

Supplementary Information 2.

Supplementary Video S1.

Supplementary Video S2.
