# Supplementary material for: Effect of pitch range on dogs’ response to conspecific vs. heterospecific distress cries
Source: Sci Rep. 2021 Oct 5;11:19723. doi: 10.1038/s41598-021-98967-w (PMC8492669; doi:10.1038/s41598-021-98967-w)
Supplement: Supplementary file 1 — Supplementary Information 1. [file 41598_2021_98967_MOESM1_ESM.docx]

**Electronic Supplementary Materials: Demographic details of dogs and Mann-Whitney U test results.**

**ESM Table 1: Demographic information for name, breed, age, sex, neuter status, experience with babies, and experience with puppies of the dogs involved in the study, as well as whether they heard babies crying in distress caused by being in the bath or following routine vaccination.**

| # | Name | Breed | Age (years) | Sex | Neutered? | Experience with babies | Experience with puppies | Condition |
| --- | --- | --- | --- | --- | --- | --- | --- | --- |
| 1 | Bertie | Cockapoo | 1 | male | No | No experience | No experience | Discomfort (bath) |
| 2 | Cassie | Terrier | 8 | female | Yes | No experience | No experience | Discomfort (bath) |
| 3 | Harriet | Collie | No data | female | No data | No data | No data | Discomfort (bath) |
| 4 | Herbie | Poodle Mix | 4 | male | Yes | No experience | Little experience | Discomfort (bath) |
| 5 | Iggy | Golden Retriever | 6 | male | No | Lots of experience | Little experience | Discomfort (bath) |
| 6 | Jasper | Labrador Mix | 3 | male | Yes | No experience | Little experience | Discomfort (bath) |
| 7 | Meeru | Labrador | 5 | female | Yes | Lots of experience | Moderate experience | Discomfort (bath) |
| 8 | Molly | Poodle Mix | 4 | female | Yes | Lots of experience | No experience | Discomfort (bath) |
| 9 | Rachel | Greyhound x whippet | 0.8 | female | No | No experience | Little experience | Discomfort (bath) |
| 10 | Rusty | Retriever Mix | 9 | male | No | Lots of experience | Little experience | Discomfort (bath) |
| 11 | Teddy | Shih Tzu | 4 | male | Yes | No experience | Little experience | Discomfort (bath) |
| 12 | Troy | Greyhound | 9 | male | Yes | No experience | No experience | Discomfort (bath) |
| 13 | Angus | Vizsla | 7 | male | Yes | Lots of experience | Little experience | Post-vaccination |
| 14 | Charlie | Miniature Schnauzer | 3 | male | No | No experience | Lots of experience | Post-vaccination |
| 15 | Devon | Spaniel | 10 | male | No | No experience | Little experience | Post-vaccination |
| 16 | Emma | Terrier | 4 | female | Yes | Lots of experience | No experience | Post-vaccination |
| 17 | Gus | Terrier Mix | 12 | male | Yes | Lots of experience | No experience | Post-vaccination |
| 18 | Hugo | Cockapoo | 2 | male | Yes | No experience | Little experience | Post-vaccination |
| 19 | Lola | Puggle | 2 | female | Yes | Little experience | No experience | Post-vaccination |
| 20 | Louis | Jack Russell x Chihuahua | No data | male | No data | No data | No data | Post-vaccination |
| 21 | Mu | Mongrel | 2 | male | No | Lots of experience | Moderate experience | Post-vaccination |
| 22 | Nova | Pug | No data | male | Yes | Lots of experience | No experience | Post-vaccination |
| 23 | Percy | Cockapoo | 2 | male | No | No experience | No experience | Post-vaccination |
| 24 | Pippa | Terrier | 7 | female | Yes | No experience | Lots of experience | Post-vaccination |
| 25 | Tilly | Labrador | 9 | female | Yes | Lots of experience | No experience | Post-vaccination |

**ESM Table 2 Mann Whitney U-test results for fundamental frequency (F0) variables varying between species across cries: Ranks.**

| **Ranks** | | | | |
| --- | --- | --- | --- | --- |
|  | Species | N | Mean Rank | Sum of Ranks |
| MeanF0 | Human baby | 12 | 6.50 | 78.00 |
|  | Puppy | 6 | 15.50 | 93.00 |
|  | Total | 18 |  |  |
| MaxF0 | Human baby | 12 | 6.50 | 78.00 |
|  | Puppy | 6 | 15.50 | 93.00 |
|  | Total | 18 |  |  |
| MinF0 | Human baby | 12 | 6.58 | 79.00 |
|  | Puppy | 6 | 15.33 | 92.00 |
|  | Total | 18 |  |  |
| Range | Human baby | 12 | 7.17 | 86.00 |
|  | Puppy | 6 | 14.17 | 85.00 |
|  | Total | 18 |  |  |
| Std Dev | Human baby | 12 | 6.58 | 79.00 |
|  | Puppy | 6 | 15.33 | 92.00 |
|  | Total | 18 |  |  |
| Large inflex | Human baby | 12 | 9.13 | 109.50 |
|  | Puppy | 6 | 10.25 | 61.50 |
|  | Total | 18 |  |  |
| Slope | Human baby | 12 | 6.67 | 80.00 |
|  | Puppy | 6 | 15.17 | 91.00 |
|  | Total | 18 |  |  |
| CofV | Human baby | 12 | 7.67 | 92.00 |
|  | Puppy | 6 | 13.17 | 79.00 |
|  | Total | 18 |  |  |
| Voice breaks | Human baby | 12 | 7.00 | 84.00 |
|  | Puppy | 6 | 14.50 | 87.00 |
|  | Total | 18 |  |  |
| Mean harmonics-to-noise ratio | Human baby | 12 | 8.58 | 103.00 |
|  | Puppy | 6 | 11.33 | 68.00 |
|  | Total | 18 |  |  |

**ESM Table 3 Mann Whitney U-test results for fundamental frequency (F0) variables varying between species across cries: Test-statistics.**

| **Test Statistics** | | | | | | | | | | |
| --- | --- | --- | --- | --- | --- | --- | --- | --- | --- | --- |
|  | MeanF0 | MaxF0 | MinF0 | Range | Std Dev | Large inflex | Slope | CofV | Voice breaks | Mean harmonics-to-noise ratio |
| Mann-Whitney U | .000 | .000 | 1.000 | 8.000 | 1.000 | 31.500 | 2.000 | 14.000 | 6.000 | 25.000 |
| Wilcoxon W | 78.000 | 78.000 | 79.000 | 86.000 | 79.000 | 109.500 | 80.000 | 92.000 | 84.000 | 103.000 |
| Z | -3.372 | -3.372 | -3.278 | -2.622 | -3.278 | -.427 | -3.184 | -2.062 | -2.817 | -1.030 |
| Asymp. Sig. (2-tailed) | .001 | .001 | .001 | .009 | .001 | .669 | .001 | .039 | .005 | .303 |
| Exact Sig. [2*(1-tailed Sig.)] | <0.001 | <0.001 | <0.001 | .007 | <0.001 | .682 | <0.001 | .041 | .003 | .335 |

**ESM Table 4 Mann Whitney U-test results for fundamental frequency (F0) variables varying between pain conditions within human baby cries: Ranks.**

| **Ranks** | | | | |
| --- | --- | --- | --- | --- |
|  | Condition | N | Mean Rank | Sum of Ranks |
| MeanF0 | Bath | 6 | 5.17 | 31.00 |
|  | Vaccine | 6 | 7.83 | 47.00 |
|  | Total | 12 |  |  |
| MaxF0 | Bath | 6 | 5.00 | 30.00 |
|  | Vaccine | 6 | 8.00 | 48.00 |
|  | Total | 12 |  |  |
| MinF0 | Bath | 6 | 5.33 | 32.00 |
|  | Vaccine | 6 | 7.67 | 46.00 |
|  | Total | 12 |  |  |
| Range | Bath | 6 | 6.00 | 36.00 |
|  | Vaccine | 6 | 7.00 | 42.00 |
|  | Total | 12 |  |  |
| Std Dev | Bath | 6 | 6.17 | 37.00 |
|  | Vaccine | 6 | 6.83 | 41.00 |
|  | Total | 12 |  |  |
| Large inflex | Bath | 6 | 6.08 | 36.50 |
|  | Vaccine | 6 | 6.92 | 41.50 |
|  | Total | 12 |  |  |
| Slope | Bath | 6 | 5.50 | 33.00 |
|  | Vaccine | 6 | 7.50 | 45.00 |
|  | Total | 12 |  |  |
| CofV | Bath | 6 | 6.75 | 40.50 |
|  | Vaccine | 6 | 6.25 | 37.50 |
|  | Total | 12 |  |  |
| Voice breaks | Bath | 6 | 7.58 | 45.50 |
|  | Vaccine | 6 | 5.42 | 32.50 |
|  | Total | 12 |  |  |
| Mean harmonics-to-noise ratio | Bath | 6 | 8.00 | 48.00 |
|  | Vaccine | 6 | 5.00 | 30.00 |
|  | Total | 12 |  |  |
|  | | | | |

**ESM Table 5 Mann Whitney U-test results for fundamental frequency (F0) variables varying between pain conditions within human baby cries: Test-statistics.**

| **Test Statistics** | | | | | | | | | | |
| --- | --- | --- | --- | --- | --- | --- | --- | --- | --- | --- |
|  | MeanF0 | MaxF0 | MinF0 | Range | Std Dev | Large inflex | Slope | CofV | Voice breaks | Mean harmonics-to-noise ratio |
| Mann-Whitney U | 10.000 | 9.000 | 11.000 | 15.000 | 16.000 | 15.500 | 12.000 | 16.500 | 11.500 | 9.000 |
| Wilcoxon W | 31.000 | 30.000 | 32.000 | 36.000 | 37.000 | 36.500 | 33.000 | 37.500 | 32.500 | 30.000 |
| Z | -1.281 | -1.441 | -1.121 | -.480 | -.320 | -.409 | -.961 | -.241 | -1.050 | -1.441 |
| Asymp. Sig. (2-tailed) | .200 | .150 | .262 | .631 | .749 | .683 | .337 | .810 | .294 | .150 |
| Exact Sig. [2*(1-tailed Sig.)] | .240 | .180 | .310 | .699 | .818 | .699 | .394 | .818 | .310 | .180 |
