# Supplementary material for: Effect of pitch range on dogs’ response to conspecific vs. heterospecific distress cries
Source: Sci Rep. 2021 Oct 5;11:19723. doi: 10.1038/s41598-021-98967-w (PMC8492669; doi:10.1038/s41598-021-98967-w)
Supplement: Supplementary file 2 — Supplementary Information 2. [file 41598_2021_98967_MOESM2_ESM.docx]

**Electronic supplementary Materials – Video notes**

Emma_Terrier_1.mp4 shows Emma a spayed female responding to vaccinated baby cry modified to conspecific frequency (950 Hz). Here, her response is given a 4 due to her strong interest in the basket: She quickly approaches it and chooses to interact with it.

Emma_Terrier_3.mp4 shows the same dog responding to the puppy cry modified to heterospecific frequency (450 Hz). Here, her response is given a 1 due to her low interest in the basket: She briefly looks at it but then swiftly loses interest and does not approach.

Note that 1 & 3 do not indicate trial order but the randomised numbers of her videos.
